# Training community health workers to limit the transmission of monkeypox virus in India: an urgent need for action

**DOI:** 10.1097/JS9.0000000000000037

**Published:** 2023-03-03

**Authors:** Saravanan Sekaran, Gayatri Devi Ramalingam, Harini Arumugasamy, Suresh Kumar R Sekar

**Affiliations:** Departments of a Prosthodontics; b Physiology, Saveetha Dental College and Hospitals, Saveetha Institute for Medical and Technical Sciences; c Faculty of Allied Health Sciences, Dr. MGR Educational and Research Institute, Chennai, Tamil Nadu, India; d College of Natural Sciences, Arba Minch University, SNNPR, Ethiopia

*Dear Editor*,

Monkeypox (MPX) is extremely unlikely to develop into a pandemic like coronavirus disease-2019 (COVID-19), but little is known about the virus. The monkeypox virus causes a less serious sickness with symptoms resembling smallpox. The potential negative effects of a MPX outbreak on public health must be kept to a minimum, particularly given the current pandemic outbreak risk. At the time of drafting this letter, according to centre for disease control and prevention, 82,147 MPX cases are reported in more than 89 countries. The standardized mortality ratio is currently between 1 and 6 %, but it might increase to 11 % and be greater among youngsters. Poverty is linked to MPX spread and socioeconomically weaker population are at high risks and spread across both urban and rural area in India.

With 70 % of the country’s population in rural areas, limited health care access community health workers (CHWs) can help in promoting access to heath care systems and aiding psychosocial support. In the recent years, COVID-19 pandemic, a major public health emergency has reiterated the importance of existing health care systems and its resilience, acoounting the significant role of CHWs to maintain uninterrupted care. They function in a nonclinical capacity, have strong contact with underserved, low-resource communities and effective in consulting and meeting social needs of the patients. CHWs are able to break social barriers, health inequities and help in revising the clinical assessment involving both clinical and nonclinical needs of the patients.^1^ Let us not forget on how CHWs augmented the health care system to address the stigma, fear and provide accurate information on COVID-19 spread, and help people to protect themselves, and obtain access to health care in rural India.

Rural Indians are more susceptible to the significant burden of disease illness owing to limited access to health care, including difficulties with screening and diagnosis.^2^ The spread of monkeypox is unchecked in india with cases increasing each day. The nation’s economy and health care system, are just starting to recover from COVID-19-related setbacks. Monkeypox might drive India’s existing broken system to its limits.

Healthcare and Family Welfare Centres provide primary health care in rural areas. A trained primary health care team of nonphysician health workers supports each center, which can typically provide services to many people. Ayushman Bharat Program, a flagship initiative by the Indian Government aims to upgrade primary health centers. In India, more than 50,000 wellness and health centers were functioning as of November 2020 and their main goal is to establish a network of 150,000 centers by December 2022.^3^ Each center comprises of a well-trained primary health care team comprising of nonmedical health workers including social activits, nurse, and multipurpose workers. With larger number of uncertified CHWs who self-trained and learned the fundamentals while working, for instance, in nursing homes or pharmacies aid in providing frontline services.

CHWs have a strong relationship with local communities and generally consider them as doctors, approach very easily owing to any health illness. Uncertified CHWs play a crucial role and account for risk to patients owing to no formal training. Induction and training of CHWs at the earlier stage of MPX would prevent the infection spike in rural areas and curb its transmission. We belive that adequeate training and proper supervision of CHWs will enable their integration to the existing stable health care system, it is also noteworthy to mention on the sustainable funding from the government is very essential. Indian minsitry of health and family welfare has already issued guildeliens for management of MPX disease, we take the liberty of enlightening the readers on how CHWs training could curb MPX transmission.

Doctors can train the groups of 70–80 CHWs in primary health care facilities. A 1-day training program on MPX has to be conducted to educate CHWs on how to:

1. Avoid contact with animals that might be infected.
2. Prevent contact with clothes, sheet, blanket, or any materials that have been interacts with a sick animal or person.
3. Use digital technology to maintain continuity of care.
4. Keep infected people apart from others.
5. Communicate with the local primary health center to set up testing and treatment.
6. Keep a list of every patient visited by CHWs.
7. Send patients with serious symptoms to specialized higher centers in the district.
8. Put on personal protective equipment during patient care.

Apart from training of CHWs, the health care system must ensure appropriate surveillance on the following:

- Ensuring adequate health care experts to provide necessary services, striking a balance between funding for ordinary medical care and pandemic response.
- Review the allocation of the health personnel frequently, and introduce task sharing as necessary.
- Ensure that all CHWs treat patients with the same infection prevention and control safety measures and also primary health center has decontamination services available at all times from staff members who have received the necessary training.
- Determine whether critical medical and nonmedical goods are required for the provision of the necessary health services.
- To prevent stock outs, keep an eye on their usage frequently and replace it.
- Create services that support CHWs psychologically and socially (revision to the existing incentives and recognition).

Needless to mention that a strong primary health care and community health care with patient focused care is essential to build longitundinal relationship among CHWs and patients. Creating awareness programmes through CHWs to educate the general public about adverse outcomes and assist them in limiting their exposure to the virus is the main strategy for preventing MPX transmission. In addition to this, rather than timely constitution of CHWs based healcare system, planning and implementation of permanent long-term palns to support CHWs is essential and a crucial part of resilient health care systems.

## Ethical approval

Not applicable

## Sources of funding

None.

## Author contribution

Dr R.S.K., Dr S.S., Dr G.D.R. and Dr H.A. performed literature search and drafted the letter.

## Conflicts of interest disclosure

The authors declare that they have no financial conflict of interest with regard to the content of this report.

## Research registration unique identifying number (UIN)

None.

## Guarantor

None.

## Data statement

The correspondence is based exclusively on resources that are publicly available on the internet and duly cited in the ‘References’ section.

No primary data was generated and reported in this manuscript. Therefore, data has not become available to any academic repository.
